# Socioeconomic factors and regional differences in mental disorder-based disability pensioning in Finland

**DOI:** 10.1192/j.eurpsy.2022.1512

**Published:** 2022-09-01

**Authors:** T. Karolaakso, R. Autio, T. Näppilä, K. Nurmela, H. Leppänen, P. Rissanen, M. Tuomisto, S. Karvonen, S. Pirkola

**Affiliations:** 1 Tampere University, Faculty Of Social Sciences, Tampere, Finland; 2 Tampere University, Medicine And Health Technology, Tampere, Finland; 3 The Finnish Institute for Health and Welfare (THL), Health And Welfare, Helsinki, Finland

**Keywords:** Disability pension, Socioeconomic, Mental health services, Regional differences

## Abstract

**Introduction:**

Prior literature has indicated low socioeconomic status (SES) and regional differences as epidemiological risk factors for disability pension (DP) due to mental disorders.

**Objectives:**

Our studies aimed to examine these associations and differences in greater detail, with separate consideration of the risk factors for mood disorders (F30–39) and non-affective psychotic disorder (F20–29) DP.

**Methods:**

Subjects (N = 36 879) were all those granted DP due to a mental disorder for the first time between 2010 and 2015 in Finland. All the subjects were matched with three controls. Education, income and occupational status were used as measures of SES. Conditional logistic regression models were used to study SES differences. Negative binomial regression analysis was used to study the levels of DP risk in the Finnish hospital districts.

**Results:**

DP recipients had low educational and income levels and often lived alone. The risk of DP was greater in white-collar occupational groups compared with blue-collar workers. Students had the greatest risk of DP for all mental and mood disorders. Significant differences in the regional mental disorder DP risks did not appear to follow the traditional Finnish health differences.

**Conclusions:**

We found evidence of SES factors and regional variation associating with mental disorder-related severe loss of working and studying ability in a disorder-specific way. The increased risk of white-collar worker DP could be related to the psychosocially demanding contemporary working life. Regional variation in DP may at least partly relate to differences in regional mental health service systems.

**Disclosure:**

No significant relationships.

